# Linking Temporal Changes in Bacterial Community Structures with the Detection and Phylogenetic Analysis of Neutral Metalloprotease Genes in the Sediments of a Hypereutrophic Lake

**DOI:** 10.1264/jsme2.ME14064

**Published:** 2014-08-13

**Authors:** Shun Tsuboi, Shigeki Yamamura, Akio Imai, Takayuki Satou, Kazuhiro Iwasaki

**Affiliations:** 1 National Institute for Environmental Studies (NIES), 16–2 Onogawa, Tsukuba, Ibaraki 305–8506, Japan

**Keywords:** bacterial community structure, sediment cores, extracellular protease genes, ammonium increase, hypereutrophic freshwater lake

## Abstract

We investigated spatial and temporal variations in bacterial community structures as well as the presence of three functional proteolytic enzyme genes in the sediments of a hypereutrophic freshwater lake in order to acquire an insight into dynamic links between bacterial community structures and proteolytic functions. Bacterial communities determined from 16S rRNA gene clone libraries markedly changed bimonthly, rather than vertically in the sediment cores. The phylum *Firmicutes* dominated in the 4–6 cm deep sediment layer sample after August in 2007, and this correlated with increases in interstitial ammonium concentrations (*p* < 0.01). The *Firmicutes* clones were mostly composed of the genus *Bacillus*. *npr* genes encoding neutral metalloprotease, an extracellular protease gene, were detected after the phylum *Firmicutes* became dominant. The deduced Npr protein sequences from the retrieved *npr* genes also showed that most of the Npr sequences used in this study were closely related to those of the genus *Bacillus*, with similarities ranging from 61% to 100%. Synchronous temporal occurrences of the 16S rRNA gene and Npr sequences, both from the genus *Bacillus*, were positively associated with increases in interstitial ammonium concentrations, which may imply that proteolysis by Npr from the genus *Bacillus* may contribute to the marked increases observed in ammonium concentrations in the sediments. Our results suggest that sedimentary bacteria may play an important role in the biogeochemical nitrogen cycle of freshwater lakes.

Sedimentary bacteria in freshwater lakes play a vital role in the degradation and transformation of organic matter (15, 36, 40). The metabolic processes of bacteria, especially in surface sediments, strongly influence water quality in shallow lakes through nutrient cycling due to diffusional material exchange between the overlying water and interstitial water of the sediments (44, 51). Thus, studying the community structure of sedimentary bacteria is indispensable for adequately understanding microbial processes and material cycling in lakes (44).

Although phosphorus is commonly considered to be the most important limiting factor in primary production, nitrogen can frequently become more important in eutrophic lakes (9). Sediments act as a significant supplier of ammonium, one of the primary nitrogen sources for the growth of phytoplankton (8, 10), to overlying water in aquatic environments (11, 41). This sediment supply function is based not only on molecular diffusion, depending on the concentration gradient of ammonium (49), but also on the resuspension of sediment particles (31, 52). These findings suggest that the sediment release process is more important in shallow lakes because they are more susceptible to the influences of hydrodynamic conditions (22).

Two important biological processes, the deamination of nitrogenous organic matter by microbes and excretion by benthic organisms, are considered the primary sources of sedimentary ammonium (27). In the case of proteins, which are one of the main sources of organic nitrogen in sediments, ammonium is produced by the deamination of constituent amino acids derived from the hydrolysates of proteins by proteases (12). Furthermore, the hydrolysis of particulate proteins is the first step in ammonium production, and is considered a kinetic limiting reaction (5, 6). Thus, the hydrolysis of proteins (proteolysis) by extracellular proteases could be the key process in the control of ammonium concentrations in sediments. Several genera of bacteria are known to produce extracellular proteases, ranging from taxa such as *Pseudomonas*, *Vibrio*, *Proteus*, *Bacillus*, and *Clostridium*, as well as various Actinomycetales (4, 12, 25). Bacterial communities dominated by the genera *Pseudoalteromonas* and *Alteromonas*, both of which produce extracellular proteases, were recently characterized based on a culture-dependent method from an ocean sediment sample (53). Extracellular protease genes have more recently been phylogenetically and quantitatively characterized in soils (32–34, 39). However, proteolytic bacterial distributions remain largely unknown in freshwater lake sediments (28). To the best our knowledge, the proteolytic bacterial communities in freshwater lake sediments have not yet been examined at the functional gene level.

The aim of this study was to provide an insight into phylogenetic and distributional features related to proteolytic bacterial communities in the sediments of hypereutrophic freshwater lakes. We used sediment core samples collected from Lake Kasumigaura, a hypereutrophic lake in Japan, to examine temporal variations in bacterial community structures, and the relationship between these variations and proteolytic functions, based on molecular approaches.

## Materials and Methods

### Study site description and sample collection

Lake Kasumigaura, the second largest freshwater lake in Japan, is located in the eastern part of the Kanto Plain, 50 km northeast of Tokyo (35°57′–36°09′N, 140°12′–140°30′E) (Fig. S1). The lake basin is smooth and shallow, with a surface area of 171 km^2^, mean depth of 4.0 m, and maximum depth of 7.3 m (20). More than 900,000 people live in the lake’s watershed (1,577 km^2^). Land use in the watershed is 30% forest, 25% paddy fields, 25% plowed fields, 10% residential, and 10% other. The lake is hypereutrophic, with mean concentrations of chlorophyll-a, nitrogen, and phosphorus of 62 μg L^−1^, 1.1 mg L^−1^, and 0.12 mg L^−1^, respectively, as measured at the center of the lake in 2010 (42). Water blooms caused primarily by the cyanobacterium *Microcystis aeruginosa* have often been observed in this lake (47).

Sediment core samples were collected using a gravitational core sampler with an acryl tube (i.d. = 4 cm; Rigo, Tokyo, Japan) at the center of Lake Kasumigaura (36°01′57″N, 140°24′25″E) between February 2007 and December 2007 (Fig. S1). Eight cores were collected at each sampling session, and brought back to our laboratory. The eight cores were cut into six sections: 0–1 cm, 1–2 cm, 2–4 cm, 4–6 cm, 6–8 cm and 8–10 cm, and then composited at each depth under N_2_-purged conditions.

After the cores were sliced at each depth, the sediment sections were transferred to thoroughly washed, N_2_-purged centrifuge tubes. The samples were then centrifuged (RCF, 2,300×*g*) for 15 min at 4°C to separate sediment and sediment pore water. The collected sediment was immediately frozen and stored at −30°C until further analysis. The sediment pore water was filtered through a pre-combusted (for 4 h at 450°C) GF/F glass fiber filter under an N_2_-purged atmosphere. The filtrate was immediately diluted by a factor of 10 for nutrient analysis. The samples were frozen and stored at −30°C until further analysis.

### DNA extraction and PCR detection

Total DNA was extracted from 200 μL of each sediment sample using the FastPrep instrument and FastDNA spin kit for soil (Q-Biogene, Carlsbad, CA, USA) according to the manufacturer’s protocol. Extracted DNA was PCR-amplified using the bacterial 16S rRNA gene universal primer pair, 350F and 920R (35) (Table 1). The PCR reaction was performed with an AmpliTaq PCR kit (Applied Biosystems, Carlsbad, CA, USA), GeneAmp PCR system 9700 (Applied Biosystems), and the Takara Thermal Cycler Dice Gradient (Takara Bio, Otsu, Japan). The PCR mixture consisted of GeneAmp PCR buffer, 0.05 U AmpliTaq Gold DNA polymerase, 1.5 mM MgCl_2_, 0.2 mM of each dNTP, 0.2 μM of each primer, 2 μL of template DNA, and nuclease-free water to a final volume of 10 μL. The PCR conditions used are given in Table S1. The amplified partial 16S rRNA gene was confirmed to be a single band by electrophoresis through a 1.0% (w/v) agarose gel and 0.5 mg L^−1^ ethidium bromide before the cloning procedure.

Bacterial protease genes (*apr*, *npr*, and *sub* genes) were amplified using specific primer pairs (3) (Table 1). Takara Ex Taq (Takara Bio) polymerase was used to amplify the protease genes. The PCR mixture included the Takara Ex Taq buffer with MgCl_2_, 0.2 mM of each dNTP, and the respective primer pairs for the target genes. PCR conditions for the three protease genes are summarized in Table S1. Successful PCR amplifications were confirmed by electrophoresis through a 2.0% (w/v) agarose gel with 0.5 mg L^−1^ ethidium bromide.

### Clone library construction, sequencing, and phylogenetic analysis

Amplified 16S rRNA and *npr* genes were cloned into the pMD20-T vector with a Mighty TA-cloning kit (Takara Bio) according to manufacturer’s protocol (for *npr*, the clone libraries were constructed using the August 4–6 cm sample only). Primer pairs and PCR conditions for the clone library construction and sequencing are summarized in Table 1 and Table S1. The constructed vectors were transformed into *Escherichia coli* JM109 competent cells (Takara Bio). Transformed *E. coli* JM109 was cultured on Luria-Bertani plates containing 100 μg mL^−1^ ampicillin, 5-bromo-4-chloro-3-indolyl-β-d-galactopyranoside (X-gal), and isopropyl-β-d-thiogalactopyranoside (IPTG) at 37°C overnight, and characterized using blue-white selection. The white colonies were checked using direct PCR with the vector primers, M13 primer M4, and M13 primer RV (Table 1), and Quick Taq HS DyeMix (TOYOBO, Osaka, Japan) to determine whether they had an insert fragment of the correct size. More than 80 random *E. coli* JM109 colonies with a PCR fragment of the correct size were picked from each sediment sample, and used for further sequencing analysis. The positive fragments were sequenced using a BigDye Terminator kit v.3.1 (Applied Biosystems), with the above vector primers, on an Applied Biosystems 3730 DNA Analyzer (Applied Biosystems).

A hierarchical taxa assignment was estimated for the sample 16S rRNA gene sequences using the Ribosomal Database Project II Classifier (http://rdp.cme.msu.edu/classifier/classifier.jsp). We used BLASTx (1) to perform a homology search of the cloned *npr* gene sequences against the GenPept database at the National Center for Biotechnology Information (NCBI). The recovered NCBI Npr protein sequences and other similar M4 protein family members were aligned to our translated *npr* gene sequences using ClustalW. The M4 family members used in the alignment were selected based on the amino acid identities of their primer binding positions (Fig. S5). An Npr sequence phylogenetic tree was estimated using the neighbor-joining method with the MEGA 5 software package (46). Bootstrap resampling analysis (1,000 replicates) was carried out to estimate the confidence of the tree topology.

### Real-time quantitative PCR (qPCR) assay

The 16S rRNA gene copy number was quantified in several sedimentary samples. Standard samples for 16S rRNA gene quantification were constructed from *E. coli* JM109 genomic DNA and its PCR products, which were amplified using the 27F and 1392R primers (2). The primer pair and PCR conditions for qPCR are summarized in Table 1 and Table S1. The standard samples produced by PCR were purified using the PureLink Quick PCR Purification kit (Invitrogen, San Diego, CA, USA) and a single band was confirmed by electrophoresis through a 2.0% (w/v) agarose gel and 0.5 mg L^−1^ ethidium bromide. The concentrations and copy numbers of standard DNA samples were measured and calculated using the Quant-it dsDNA Broad-Range assay kit and Qubit Fluorometer (Invitrogen) according to the manufacturer’s protocol. qPCR was carried out using a Thermal Cycler Dice Real Time System Single (Takara Bio) and MightyAmp for Real Time (SYBR Plus) (Takara Bio) according to the manufacturer’s protocol. All analyses were carried out in triplicate on each extracted DNA sample. The qPCR amplification efficiency and correlation coefficient (R^2^) of the standard curve were 85.0% and 0.99, respectively.

### Analytical methods

The concentrations of ammonium (NH_4_-N), dissolved total nitrogen (DTN), and orthophosphate (PO_4_-P) in the pore water samples were measured with an auto analyzer (Traacs 800, Bran + Luebbe, Tokyo, Japan); the concentration of dissolved organic carbon (DOC) was calculated with a Shimadzu TOC-5000 total organic carbon analyzer equipped with a Pt catalyst on quarts wool (17, 20). These data were previously reported in a NIES Research Project Report (37) and were primarily used to describe the distance-based redundancy analysis (db-RDA) of the relationships between bacterial community structures and environmental variables. These results are presented in Figs. S2 and S3a.

### Statistical analysis

A distance-based redundancy analysis (db-RDA) was performed using “R” statistics software (R Development Core Team, version 2.15.2) within the “vegan” package to study the relationship between each phylogenetic phylum in the bacterial community and environmental variables (38). Data for each phylogenetic phylum were assigned as the relative abundances of the detection frequency for each clone library.

### Nucleotide sequence accession numbers

The nucleotide sequences of the partial 16S rRNA and *npr* genes obtained in this study have been deposited into the DDBJ/EMBL/ GenBank databases under the following accession numbers: AB928631 through AB930045 for the 16S rRNA genes, and AB930046 through AB930120 for the *npr* genes.

## Results

### Spatial and temporal variations in bacterial community structures

**Fig. 1:**
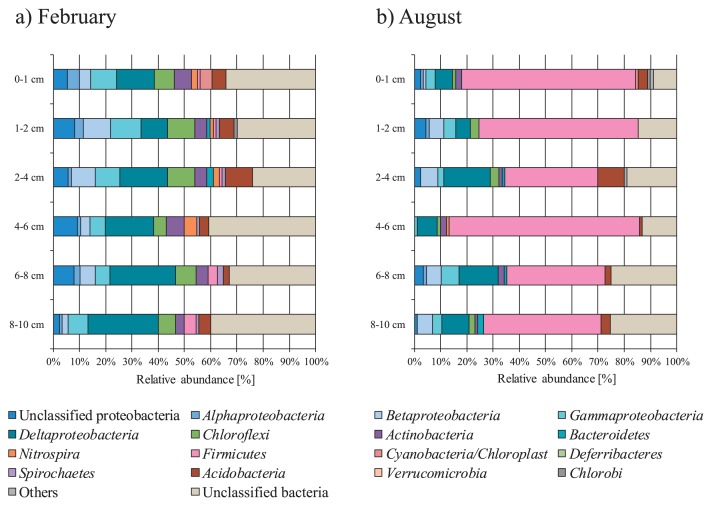
Vertical variations in bacterial community structures determined from the 16S rRNA gene clone libraries of sediment core samples collected from Lake Kasumigaura in a) February and b) August 2007.

**Fig. 2:**
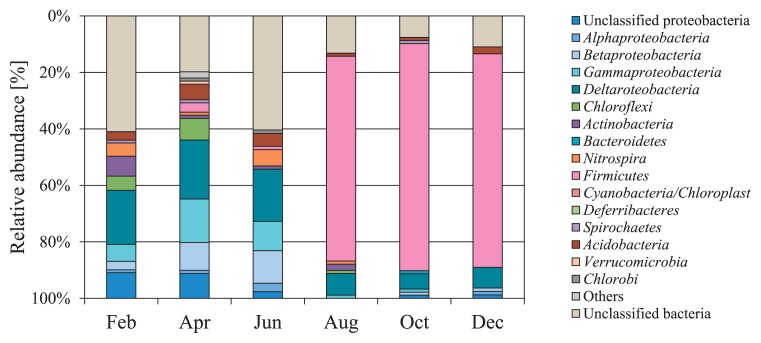
Bimonthly temporal variations in bacterial community structures determined from 16S rRNA gene clone libraries of 4–6 cm deep sediment core samples collected in 2007.

The structures and compositions of the bacterial communities in the sediment core samples were analyzed by preparing 16S rRNA gene clone libraries. Vertical variations in bacterial community structures between the February and August 2007 samples are shown in Fig. 1. Apparent vertical variations were not observed in the two sediment cores. However, the composition of these bacterial communities clearly differed between the February and August cores. In February, in order of prevalence, *Deltaproteobacteria* (10.3–26.7%), *Gammaproteobacteria* (5.7–11.5%), *Chloroflexi* (4.7–10.3%), *Acidobacteria* (2.3–10.3%), *Betaproteobacteria* (2.2–10.3%), and *Actinobacteria* (3.3–7.0%) were dominant. However, in August, the phylum *Firmicutes* dominated all depths (35.6– 72.5%). Temporal variations in bacterial community structures in the 4–6 cm deep samples are shown in Fig. 2. Marked changes were observed in bacterial community structures changed after August. The phylum *Proteobacteria* was dominant between February and June. In contrast, the phylum *Firmicutes* accounted for 72.5% to 80.4% of 16S rRNA gene clones between August and December. The majority of *Firmicutes* observed (65.2% in August, 79.7% in October, and 83.9% in December) were classified into the genus *Bacillus*, as estimated by the Ribosomal Database Project classifier with an 80% confidence threshold.

### Spatial and temporal variations in the measured environmental variables in pore water

Dissolved organic carbon (DOC), ammonium (NH_4_-N), orthophosphate (PO_4_-P), and dissolved total nitrogen (DTN) were measured in pore water samples collected in February and August in 2007 (Fig. S2). The concentration ranges of DOC, NH_4_-N, PO_4_-P, and DTN were 4.6–6.4 mg L^−1^, 1.7–5.9 mg L^−1^, 0.07–0.65 mg L^−1^, and 2.5–7.3 mg L^−1^, respectively. All the measured variables were slightly higher in August than in February. Temporal variations in DOC, NH_4_-N, PO_4_-P, and DTN at the 4–6 cm depth are shown in Fig. S3a. NH_4_-N and DTN concentrations markedly increased after April from 3.4 mg L^−1^ to 8.3 mg L^−1^ and from 4.5 mg L^−1^ to 9.4 mg L^−1^, respectively. These results indicated that ammonium accounted for a large portion of the DTN in the pore water of the sediment samples examined in the present study.

### Relationships between bacterial community structures, 16S rRNA gene copy numbers, and environmental variables

**Fig. 3:**
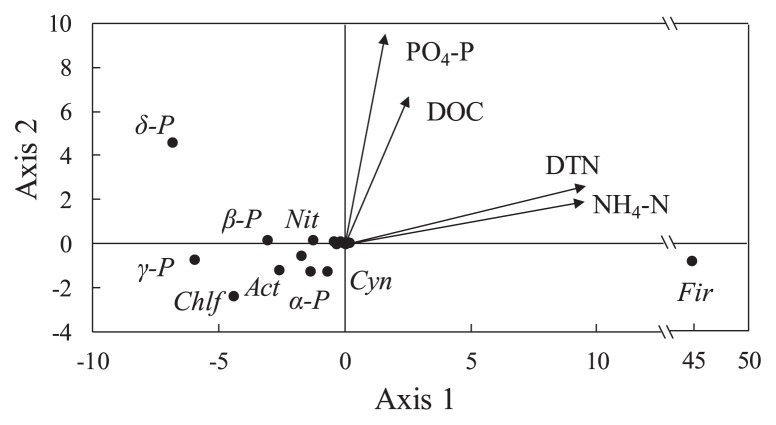
Distance-based redundancy analysis between bacterial community structures in sediment determined from 16S rRNA gene clone libraries and environmental variables measured in the pore water of the sediment. Circles indicate the main respective bacterial phyla or subdivisions in the case of *Proteobacteria*. Arrows indicate environmental variables used. Abbreviations used: *Act*, *Actinobacteria*; *α-P*, *Alphaproteobacteria*; *β-P*, *Betaproteobacteria*; *γ-P*, *Gammaproteobacteria*; *δ-P*, *Deltaproteobacteria*; *Chlf*, *Chloroflexi*; *Cyn*, *Cyanobacteria/Chloroplast*; *Fir*, *Firmicutes*; *Nit*, *Nitrospira*; DOC, dissolved organic carbons; DTN, dissolved total nitrogen; NH_4_-N, ammonium nitrogen; PO_4_-P, orthophosphate.

A distance-based redundancy analysis (db-RDA) was used to evaluate relationships between bacterial community structures and environmental variables (Fig. 3). DTN and NH_4_-N positively correlated with the relative clone ratios of the phylum *Firmicutes* in the 16S rRNA gene clone libraries. A permutation test indicated that the NH_4_-N concentration was the most significant factor controlling the bacterial community structures in the sediment (*p* < 0.01).

Bacterial 16S rRNA gene copy numbers were quantified in the sediment samples at the 4–6 cm depth in 2007 (Fig. S3b). The copy numbers ranged from 2.25×10^11^ to 4.64×10^11^ copies mL^−1^ sediment, indicating that bacterial abundances hardly varied at a depth of 4–6 cm during the sampling period, and that the increase observed in NH_4_-N concentrations did not directly influence the abundance of sedimentary bacteria.

### Distributional and molecular characteristics of genes encoding three extracellular proteolytic enzymes

Three extracellular proteolytic enzyme genes, alkaline metalloprotease (*apr*), thermolysin-like neutral protease (*npr*), and subtilisin-like serine protease (*sub*), were analyzed in the sediment samples at the 4–6 cm depth bimonthly between February and December 2007 (Fig. S4). *npr* genes were detected after August; however, *apr* and *sub* genes were not amplified in any sample.

**Fig. 4:**
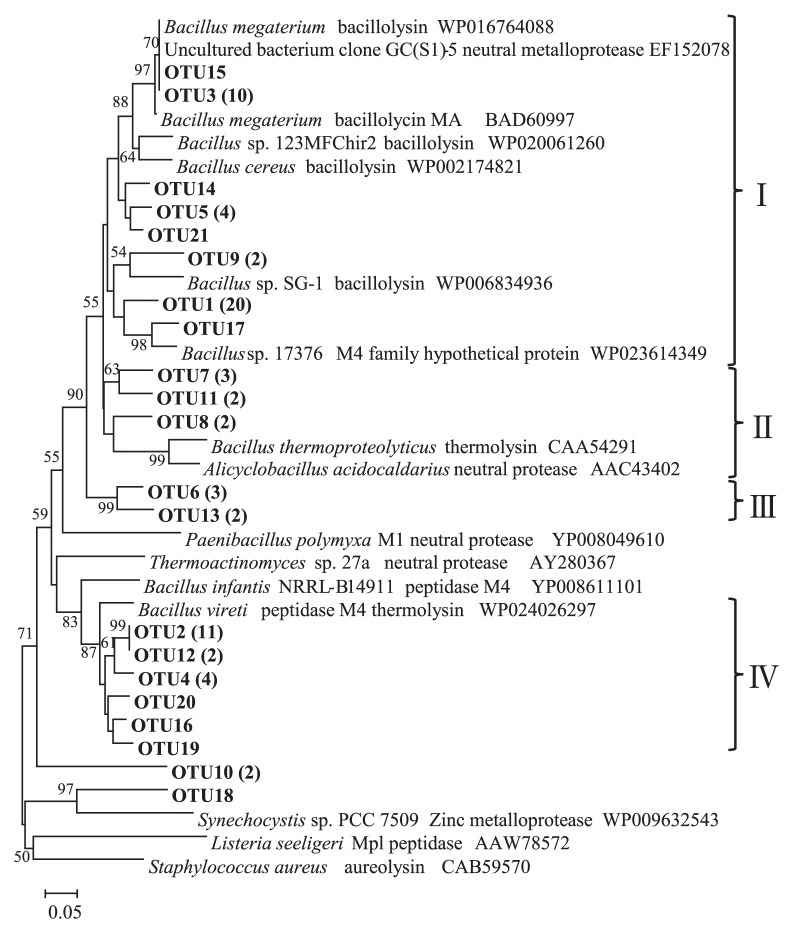
Neighbor-joining phylogenetic tree of the deduced Npr sequences retrieved from the 4–6 cm deep sediment sample in August (bold font), *npr*-related environmental clones from a previous study (34), and reference sequences of Npr-related M4 family proteins from GenPept. Values in parenthesis indicate the number of sequences in each OTU. Bootstrap values greater than 50% based on 1,000 replicates are shown at the nodes.

**Fig. 5:**
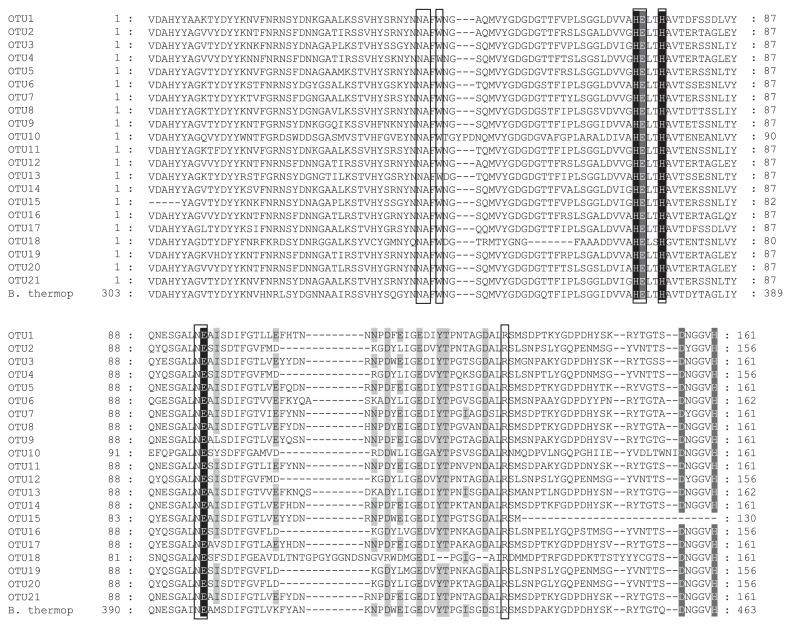
Alignment of amino acid sequences translated from cloned *npr* genes obtained from the 4–6 cm deep sediment sample in August. Each OTU displays a representative sequence for that particular OTU. White letter residues shaded black indicate zinc binding sites. White letter residues shaded dark gray indicate catalytic triads. Black letter residues shaded light gray indicate calcium binding sites. Boxes highlight catalytic amino acid residues. “B. thermop” indicates the thermolysin sequence of *Bacillus thermoproteolyticus* (CAA54291).

We amplified *npr* genes using PCR primers designed from the *npr* gene sequences of 61 organisms by Mrkonjic Fuka *et al.* (34). Our literature review indicated that this primer set may be able to detect not only *npr*-related genes, but also some of the other M4 family proteins (Fig. S5). However, of the 75 cloned genes retrieved from the 4–6 cm deep sediment samples in August, 74 of the clones exhibited high similarities, ranging between 61% and 100%, with known Npr sequences from the genus *Bacillus* at the amino acid level based on a BLASTx search of the NCBI GenPept database. The 75 Npr-related sequences obtained were divided into 21 OTUs (operational taxonomic units) using a 90% similarity cut-off value at the nucleic acid level. A phylogenetic tree of the conserved amino acid sequences is shown in Fig. 4. OTU1, OTU2, and OTU3 included 20, 11, and 10 of the clone members, respectively. Almost all of the Npr sequences obtained in the present study were assigned to four clusters. Approximately half of the clones (53.3%; 40/75) were designated as cluster I along with several bacillolysin-related Npr sequences belonging to the genus *Bacillus*. Cluster II clones (9.3%; 7/75) were closely related to *Bacillus thermoproteolyticus* thermolysin (CAA54291) and *Alicyclobacillus acidocaldarius* neutral protease (AAC43402). Cluster III (6.7%; 5/75) formed a specific branch distinct from the Npr reference sequences. Cluster IV, the second major group of our clones (26.7%; 20/75), also contained Npr-related sequences from the genus *Bacillus*, but its phylogenetic position was apparently separate from cluster I, though its basal bootstrap support was weak. The representative amino acid sequences of each sample OTU, aligned to that of the known neutral metalloprotease from *Bacillus thermoproteolyticus*, highlighted common Npr motifs, including catalytic amino acids, and zinc and calcium binding sites, as shown in Fig. 5.

## Discussion

Vertical variations in bacterial community structures in the sediments of freshwater lakes have been examined in many studies (21, 24, 48, 51). As a general trend, any vertical shift in the bacterial community structures of sediments in eutrophic lakes is minor. Li *et al.* (24) suggested that the minimal vertical variations observed in bacterial community compositions may be attributed to an abundance of nutrients in the sediments. In our study, we observed similar minimal vertical variations in bacterial community structures, although marked changes were observed bimonthly in their compositions changed (Fig. 1). This result suggested that bacterial communities synchronously and markedly change their compositions over time, at least in the upper 10 cm of the sediment cores evaluated in this study.

At the 4–6 cm depth, the bacterial community structures were similar from February through to June, but markedly changed after August when *Firmicutes*-related clones dominated (Fig. 2). Similar to our results, the dominance of the phylum *Firmicutes* has been reported in previous studies freshwater lake sediments (43, 45). Song *et al.* (43) demonstrated that the phylum *Firmicutes* temporarily dominated at a sampling site around the river mouth of Dongping lake due to significant amounts of allochthonous inputs, including soils, particulate organic matter, and fertilizers in the lake. In contrast, in our study, the upper 2 cm of the sediments at the center of Lake Kasumigaura took approximately 5.2 years to accumulate (20). Thus, the dominance of the phylum *Firmicutes* after August was not likely to have been due to an external input, but rather to sediment variations inside the column. In the phylum *Firmicutes*, the genus *Bacillus* accounted for 65.2% (at the 4–6 cm depth in August) to 83.9% (at the 4–6 cm depth in December), although the copy numbers of bacterial 16S rRNA gene per mL^−1^ of sediments changed little in the sediment samples at the 4–6 cm depth during the sampling period (Fig. S3b). The genus *Bacillus* is a bacterial r-strategist (26), which has a higher maximum growth rate (13), and is able to rapidly adapt to changes in environmental conditions (19). The marked increase observed in the relative contribution of the genus *Bacillus* in the present study suggests that this genus is of considerable ecological importance to the sediments.

A marked increase was also observed in ammonium concentrations in the pore water of Lake Kasumigaura during our bimonthly sampling. This particular phenomenon has not been reported previously in other freshwater lakes. Extracellular proteases may play a vital role in increasing NH_4_-N concentrations in the pore water of sediments because proteins are the primary fraction of nitrogenous organic matter in sediments. There are primarily three bacterial extracellular protease enzymes: alkaline metalloprotease (Apr), neutral metalloprotease (Npr), and serine protease (Sub) (16). Apr has a broad specificity, an optimum pH of 7–9, and is produced by bacteria including the genera *Pseudomonas* and *Serratia*. Npr has a substrate preference for hydrophobic or large amino acid residues, with an optimum pH near 7. This protease has been observed across a broad range of taxa, including the genus *Bacillus* in bacteria and *Aspergillus* in fungi. Sub is a representative subtilisin produced by *Bacillus subtilis*. In our study, the *npr* gene was detected in the sediment cores after August when *Bacillus*-related 16S rRNA gene clones dominated (Fig. S4). Mrkonjic Fuka *et al.* (34) detected *npr* genes related to a broad range of bacterial taxa, including the genera *Vibrio*, *Bacillus*, *Paenibacillus*, *Clostridium*, *Thermoactinomyces*, and *Alicyclobacillus*, in soil using a clone library method. In contrast, 74 of the retrieved 75 Npr-related sequences from 4–6 cm deep sediment core sections in August were phylogenetically affiliated to the Npr-related proteins of the genus *Bacillus* in our study. Sakurai *et al.* (39) also reported that most of the *npr* genes they collected in soils, based on denaturing gradient gel electrophoresis, were consistent with *Bacillus*-like *npr* genes. Moreover, the pore water NH_4_-N concentration in the sediments correlated with the frequency of the occurrence of the phylum *Firmicutes* in our clone library of 16S rRNA gene sequences (*p* < 0.01, Fig. 3). Therefore, our results suggest that proteolysis by *Bacillus* Npr proteins may perform important ecological functions by markedly increasing NH_4_-N concentrations in the pore water of sediments in hypereutrophic lakes.

Ecologically functional features associated with the *npr* gene may determine and characterize proteolysis in aquatic sediments to a substantial degree. The deduced amino acid sequences from the *npr* genes we recovered showed the specific amino acid residues and zinc-binding sites (18, 23) necessary for neutral metalloprotease activity (Fig. 5). Furthermore, specific calcium-binding sites, which play an important role in stabilizing conformations by preventing autolysis (29), were also detected (Fig. 5) in our sequences. These results contribute to the argument that sedimentary proteolytic activity is affected by variations in the redox potential of sediments, which controls heavy metal availability (30). Jones and Grant (14) suggested that alkaliphilic *Bacillus* spp. play a significant role in the decomposition of biopolymers, including the proteins, and celluloses and other complex carbohydrates in the sediments of soda lakes. If Npr proteins have a vital role in the proteolysis of sediments, studies regarding the gene expression and activity of Npr proteins may shed light on the unknown ecological roles of the genus *Bacillus* in the decomposition of proteins as well as the turnover of organic nitrogen in hypereutrophic freshwater lake sediments.

Npr is a protein member of the M4 family, which comprises metalloendoproteases that bind a single zinc ion and are active at a neutral pH (MEROPS database, http://merops.sanger.ac.uk/cgi-bin/famsum?family=M4). Many M4 family proteins are also recognized pathogenicity factors (50). The majority of the Npr sequences obtained in this study (more than 50%) are phylogenetically related to bacillolysin (Fig. 4), which is an Npr-related protein from the genus *Bacillus*. Chung *et al.* (7) suggested that bacillolysin may be responsible for the pathogenicity of the genus *Bacillus*. Therefore, understanding the occurrence of the *npr* gene may also be important in revealing the risks associated with human health in hypereutrophic freshwater lakes utilized as raw drinking water sources.

## Conclusions

In the present study we detected *npr*-related genes in the sediments of a hypereutrophic lake, and found that they were associated with the dominance of the genus *Bacillus* and also with a marked increase in interstitial NH_4_-N concentrations. These results implied that proteolysis by sedimentary bacteria may contribute to the production of NH_4_-N in sediment pore water, and may ultimately play an important role in supplying nitrogen from the sediment to the overlying water. Further studies (*e.g.*, gene expression and Npr enzymatic activity assays) are required to more clearly determine the ecological contributions of sedimentary bacteria toward the biogeochemical cycling of nitrogen in freshwater lakes.

## Supplemental materials



## Figures and Tables

**Table 1:** PCR primers used for this study

Target gene	Primer name	Sequence (5′ to 3′)	Use for	Reference
*apr*	FP apr I	TAYGGBTTCAAYTCCAAYAC	Detection	(3)
	RP apr II	VGCGATSGAMACRTTRCC	Detection	

*sub*	FP sub I	ATGSAYRTTRYYAYATGAG	Detection	(3)
	RP sub II	GWGWHGCCATNGAYGTWC	Detection	

*npr*	FP npr I	GTDGAYGCHCAYTAYTAYGC	Detection/Cloning	(3)
	RP npr II	ACMGCATGBGTYADYTCATG	Detection	
	RP npr IIb	RTGDACNCCDCCRYWRTC	Cloning	(34)

16S rRNA	27F	AGAGTTTGATCMTGGCTCAG	qPCR standard construction	(2)
	350F	CCTACGGGAGGCAGCAG	Cloning/Quantification	(35)
	920R	CCGTCAATTCCTTTGAGTTT	Cloning/Quantification	
	1392R	ACGGGCGGTGTGTAC	qPCR standard construction	(2)

Vector	M13 primer M4	GTTTTCCCAGTCACGAC	Insert check/Sequencing	Takara Bio
	M13 primer RV	CAGGAAACAGCTATGAC	Insert check/Sequencing	Takara Bio
