# Supplementary material for: Linking Temporal Changes in Bacterial Community Structures with the Detection and Phylogenetic Analysis of Neutral Metalloprotease Genes in the Sediments of a Hypereutrophic Lake
Source: Microbes Environ. 2014 Aug 13;29(3):314–21. doi: 10.1264/jsme2.ME14064 (PMC4159043; doi:10.1264/jsme2.ME14064)
Supplement: Supplementary file 1 [file 29_314_s1.pdf]

Supplemental materials

Linking temporal changes in bacterial community structure with the detection and phylogenetic analysis of neutral metalloprotease genes in the sediments of a hypereutrophic lake

Shun Tsuboi[1]*, Shigeki Yamamura[1], Akio Imai[1], Takayuki Satou[1] and Kazuhiro Iwasaki[1]

[1]Center for Regional Environmental Research, National Institute for Environmental Studies, 16-2 Onogawa, Tsukuba, Ibaraki 305-8506, Japan

* Corresponding author. Tel: +81-29-850-2407; Fax: +81-29-850-2407;

E-mail: tsuboi.shun@nies.go.jp

Table S1. PCR conditions used in this study and its objectives

Target gene	Objective	Each primer conc. (μM)	Initial denaturation		No. of cycles	Denaturing		Annealing		Extension		Final Extension	
			Temp (°C)	Time (s)		Temp (°C)	Time (s)	Temp (°C)	Time (s)	Temp (°C)	Time (s)	Temp (°C)	Time (s)
apr	Detection	4	95	300	35	94	30	52	30	72	30	72	600
sub	Detection	4	95	300	35	94	30	52	30	72	20	72	600
npr	Detection	4	95	300	35	94	30	52	30	72	20	72	600
	Cloning	4	95	300	10/35	94	30	56/46[a]	30	72	30	72	600
16S rRNA	Cloning	0.2	95	600[c]	20/20	95	15	60/50[b]	15	72	30	72	600
	qPCR standard	0.5	95	600[c]	30	94	30	55	30	72	90	72	600
	Quantification	0.2	98	120	40	98	10	60	15	68	60		

[a] The annealing temperature for the PCR decreased from 56 to 47°C at 1°C/cycle and was kept constant at 46°C for the last 35 cycles.

[b] The annealing temperature for the PCR decreased from 60 to 50°C at 1°C/two cycles and was kept constant at 50°C for the last 20 cycles.

[c] Initial denaturation time was 600 sec because AmpliTaq® Gold (Life Technologies), which needs a PCR enzyme activation process by heat, was used.

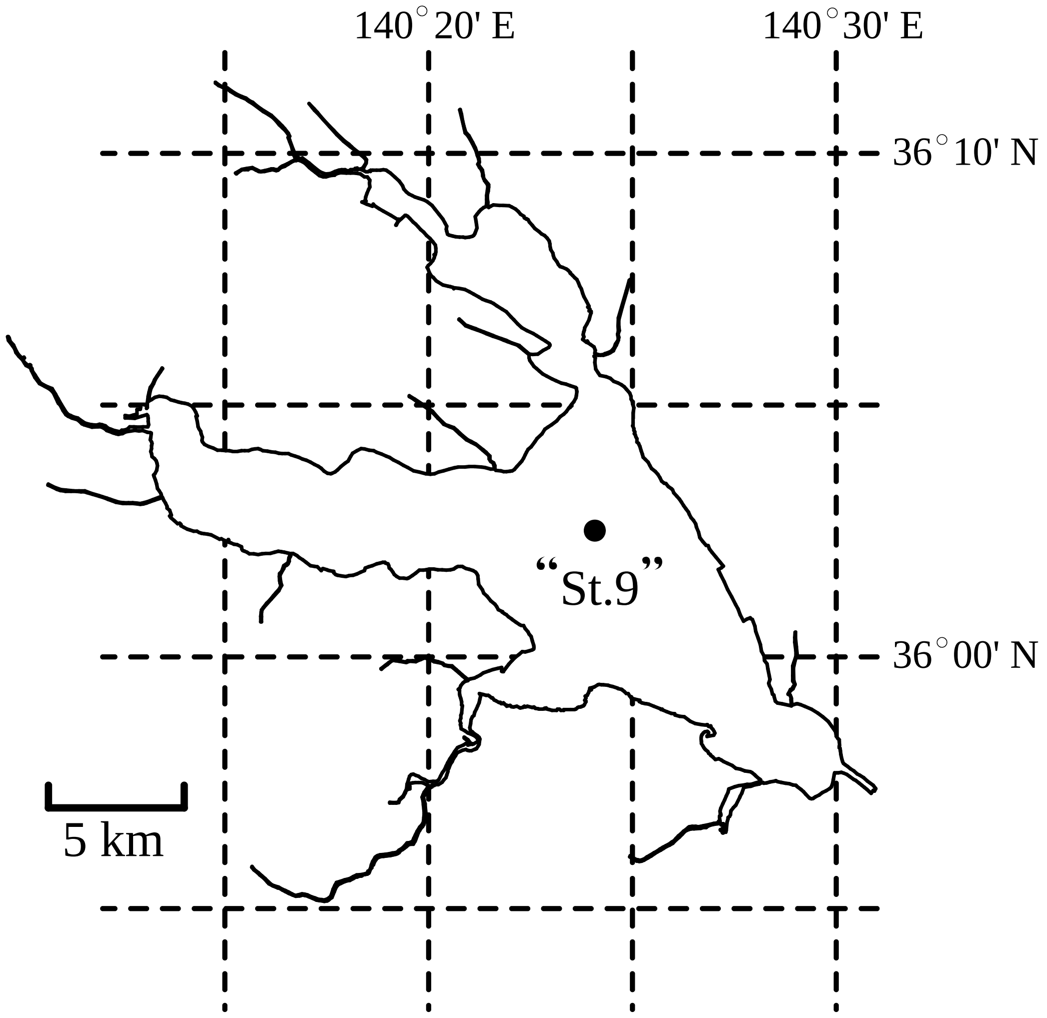

Fig. S1. Map of sampling site in this study. The sampling station indicates as "St. 9" in this map.

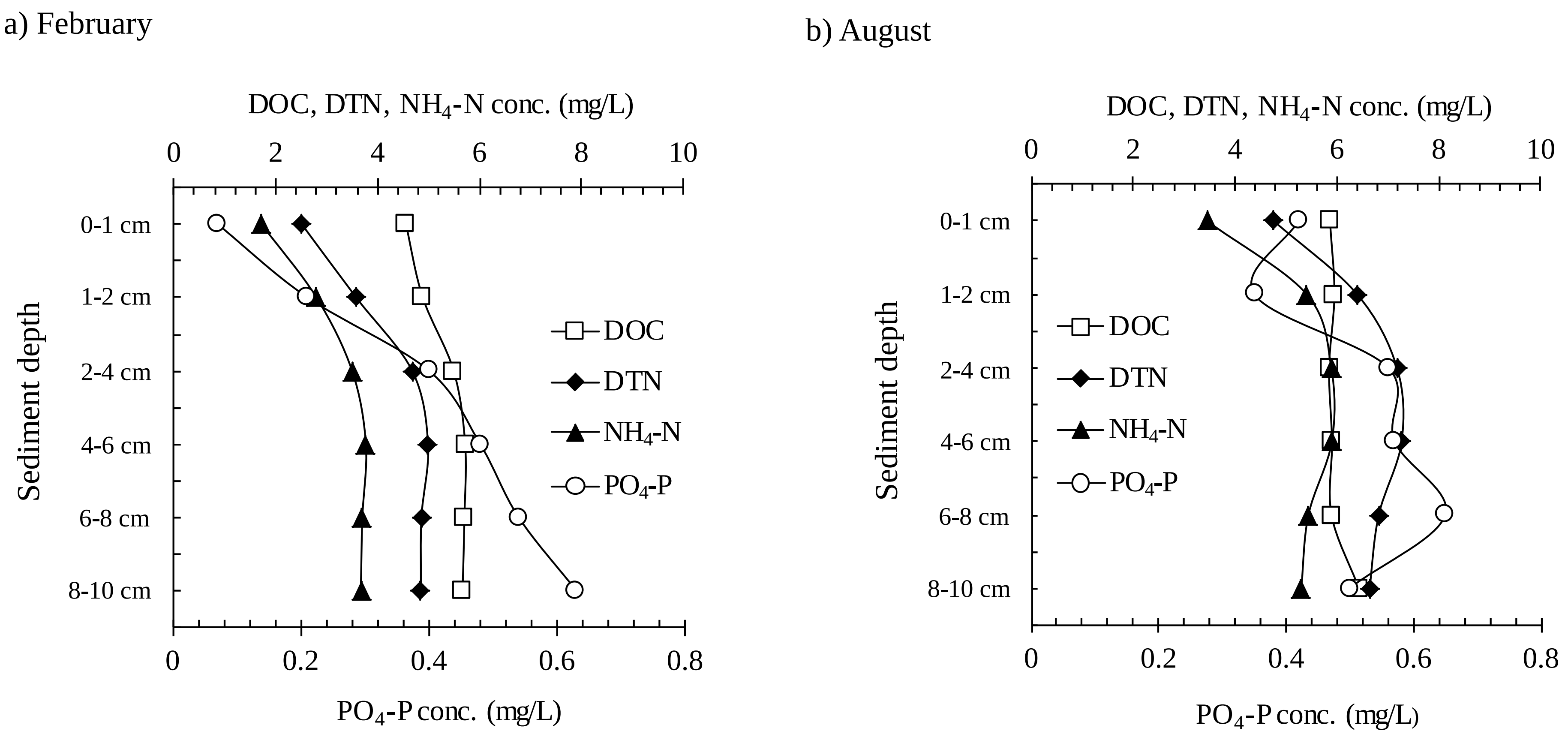

Fig. S2. Vertical variations of environmental variables in pore water of the sediment core samples. a) February and b) August.

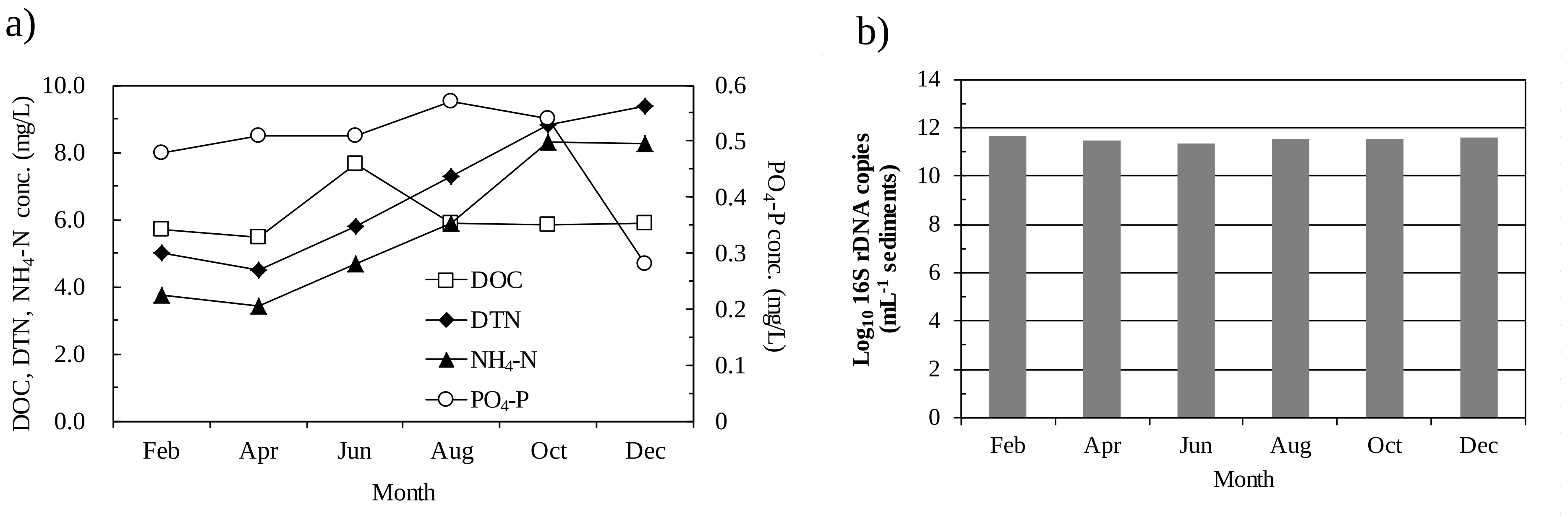

Fig. S3. Bimonthly temporal variations of a) environmental variables and b) 16S rRNA gene copy numbers per sediment mL^{-1} in 4–6 cm depth sediments.

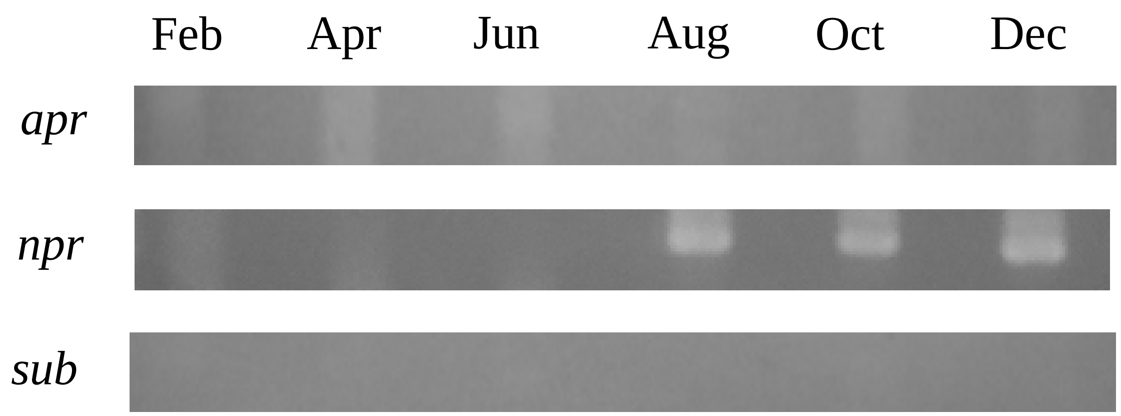

Fig. S4. Detection of protease genes in bimonthly samples of 4–6 cm depth in 2007. *apr*: alkaline metalloprotease gene, *npr*: neutral metalloprotease gene, *sub*: serine protease gene.

Vibrio proteolyticus vibriolysin AAA27548	NDAHYFGKV	-DYYNGLNV
Pseudomonas aeruginosa PAO581 pseudolysin AGV61519	NDAHFFGGV	-QYYNGIDV
Legionella oakridgensis RV-2-2007 Msp peptidase ETO93853	NDALYAGMI	-EYYSGLDV
Vibrio sp. T-1800 vimelysin BAC87681	NDAHYFGNV	-QYYDGLNV
Aeromonas hydrophila elastase AAF07184	NDAHYFGNV	-DYYDGIDV
Streptomyces griseus NBRC 13350 griselysin YP001823607	ADAHYGAAE	---IGNVDV
Pseudoalteromonas piscicida MprIII BAC22656	NDAHHHGQT	-DYDDSVTS
Vibrio harveyi pap6 peptidase AAM34261	NDAHYFGQR	-QYYRGIDV
Burkholderia lata ZmpA peptidase YP366852	ADIDYGLAL	SWSNPRHDP
Burkholderia cenocepacia J2315 zinc metalloprotease ZmpB YP002234906	VNVYYHIDT	RTYAQARGAE
Bacillus sp. PPB15 neutral protease B ADR72651	VDAHYNAQK	TGTADNGGVH
Cronobacter sakazakii zinc metalloprotease ABK56826	DEAYDYLGV	--REDNGGVH
Photorhabdus sp. Az29 PrtS peptidase ABY26041	DNAYKYLEA	-IYRDNGGVH
Serratia grimesii protealysin ABY40626	DEAYDYLGV	--KEDNGGVH
Enterococcus faecalis V583 coccolysin NP815516	VDAYTHGKF	TPYYDQGGVH
Clostridium perfringens lambda toxin CAD27906	VSAHSYAGV	TENGDYGGVH
OTU1	VDAHYYAAK	TGTSDNGGVH
Bacillus megaterium bacillolysin MA BAD60997	VDAHYYAGV	TGSSDNGGVH
Bacillus thermoproteolyticus thermolysin CAA54291	VDAHYYAGV	TGTQDNGGVH
Listeria seeligeri Mpl peptidase AAW78572	VDAHYYAGE	TEDDDWGGIH
Staphylococcus aureus aureolysin CAB59570	VDANYYAKQ	TEK-DNGGVH
Paenibacillus polymyxa M1 neutral protease YP008049610	VDAHAYAAK	RGSSDNGGVH
Alicyclobacillus acidocaldarius neutral protease AAC43402	VDAHYYAGV	TGTQDNGGVH
Thermoactinomyces sp. 27a neutral protease AY280367	VDAHYYAGK	TREGDWGGVH
Uncultured bacterium clone GC(S1)-5 EF152078	VDAHYYAGV	TGSSDNGGVH
Synechocystis sp. PCC 7509 Zinc metalloprotease WP009632543	AKAHTFARD	KDSDDNGGVH
Consensus sequence	VDAHYY (Forward primer)	DNGGVH (Reverse primer)

Fig. S5. Amino acid sequence alignments of primer binding sites of representative M4 family proteins. Based on this alignment, a phylogenetic tree of Npr-related proteins was constructed with the amino acid sequences of similar proteins in M4 family (Fig. 4). Protein sequences used are boxed. *Synechocystis* sp. PCC 7509 Zinc metalloprotease (WP009632543) was also used because the internal sequence was similar to the internal sequence of clone OTU18.
